# Mexenone protects mice from LPS-induced sepsis by EC barrier stabilization

**DOI:** 10.1371/journal.pone.0302628

**Published:** 2024-05-09

**Authors:** Yoon Ji Choi, Jimin An, Ji Hye Kim, Sa Bin Lee, Bo Seok Lee, Chae Young Eom, Hyohi Lee, Nayeong Kwon, Il Shin Kim, Kyoung-Su Park, Sooah Park, Jung-Woog Shin, Sanguk Yun

**Affiliations:** 1 In Vivo Research Center (IVRC), UCRF, UNIST, Ulsan, Korea; 2 Department of Biotechnology, Inje University, Gimhae, Korea; 3 Department of Biomedical Engineering, Inje University, Gimhae, Korea; Ann and Robert H Lurie Children’s Hospital of Chicago, Northwestern University, UNITED STATES

## Abstract

Blood vessels permit the selective passage of molecules and immune cells between tissues and circulation. Uncontrolled inflammatory responses from an infection can increase vascular permeability and edema, which can occasionally lead to fatal organ failure. We identified mexenone as a vascular permeability blocker by testing 2,910 compounds in the Clinically Applied Compound Library using the lipopolysaccharide (LPS)-induced vascular permeability assay. Mexenone suppressed the LPS-induced downregulation of junctional proteins and phosphorylation of VE-cadherin in Bovine Aortic Endothelial Cells (BAECs). The injection of mexenone 1 hr before LPS administration completely blocked LPS-induced lung vascular permeability and acute lung injury in mice after 18hr. Our results suggest that mexenone-induced endothelial cell (EC) barrier stabilization could be effective in treating sepsis patients.

## Introduction

Sepsis is a life-threatening disease and was responsible for 11 million deaths worldwide in 2017 [1]. In 2018, an estimated 15% of all neonatal deaths globally were due to sepsis [2]. Dysregulated host responses to infections cause systemic inflammation, damaging multiple organs and resulting in liver, kidney, lung, and brain organ failure. Septic shock due to major fluid leakage into the extravascular space and subsequent poor blood perfusion often leads to high mortality [3]. Without prompt intervention, mortality rates exceed 30% [4].

The current sepsis treatment has focused on two arms of management, including antibiotic administration for infection control and the administration of intravenous fluid or vasoconstrictor agents for hemodynamic management [4]. There also have been attempts to modulate host immune responses with anti-inflammatory drugs. However, neither glucocorticoid nor vitamin C treatment improved sepsis survival [5]. Since the withdrawal of Xigris from the market in 2011, the development of specific sepsis drugs targeting immune cell factors or cytokines has not been successful [6].

Endothelial cells line the innermost layer of the vasculature. Blood vessels transport oxygen and nutrients to peripheral tissues and, at the same time, perform a barrier function preventing dysregulated blood content leakage. Vascular permeability is an underlying cause or a major contributing factor in multiple inflammatory diseases, such as atherosclerosis, stroke, and sepsis [7]. The disruption of the endothelial cell-cell junction by endotoxin or inflammatory cytokines triggers vascular permeability in sepsis [8].

Dynamic EC junction formation or disruption is important for vascular development, angiogenesis, vascular homeostasis, and vascular inflammation. Vascular endothelial growth factor (VEGF) and inflammatory ligands induce adherens junction (AJ) disassembly via multiple pathways [9]. Endothelial adherens junctions are mediated by vascular endothelial cadherin (VE-cadherin). VE-cadherin interacts with p120-catenin, β-catenin, and ɑ-catenin, which link to the cytoskeleton and signaling molecules [10]. The integrity of adherens junctions or tight junctions (TJs) is compromised by changing the phosphorylation status of key junctional proteins, such as VE-cadherin or ZO-1 [11]. Inflammatory ligands also promote the RhoA activity, leading to actin stress fiber-dependent contractions and pore formation [12].

We screened a chemical library to identify drug candidates that could block LPS-induced vascular permeability *in vitro*. One of the hits identified in the clinically applied compound library was mexenone, which is a UV-absorbing ingredient in sunscreen agents.

## Materials and methods

### Cell culture

Bovine aortic endothelial cells (BAECs) isolated from bovine aorta were grown in Dulbecco’s modified Eagle’s medium (DMEM) with 10% fetal bovine serum (FBS) and penicillin and streptomycin. BAECs were maintained at 37°C in a 5% CO_2_ incubator. Human Lung Microvascular Endothelial Cells (HLMVECs) were purchased from Cell Biologics (USA) and grown in Complete Human Endothelial Cell Medium (Cell Biologics).

### Chemical library screening

A clinical drug library containing 2,910 compounds was provided by the Korea Chemical Bank. The library includes approved drugs and those in clinical trials. Compounds were treated at 1 μM concentration to EC for 90 min before stimulation with LPS for 3h. The compounds reducing endothelial permeability by more than 50% compared to dimethyl sulfoxide (DMSO) controls were chosen as hits.

### Reagents

Streptavidin and Flamma496 were purchased from BioActs (RFP0716). Bovine fibronectin was isolated from bovine plasma using gelatin-agarose beads as previously described [13]. Isolated fibronectin was biotinylated using the EZ-Link™ Sulfo-NHS-LC-Biotinylation Kit (ThermoFisher, USA, 21435).

**Table:** Antibodies.

Antibodies	Company, product number	Usage
p-p65	Cell Signaling Technology, USA, #3033	Fig 1D, immunoblot
ZO-1	Invitrogen, USA, #61–7300	Fig 1D, immunoblot
VE-cadherin	Abcam, USA, ab33168	Fig 1D, immunoblot
Vimentin	Cell Signaling Technology, USA, #5741	Fig 1D, immunoblot
VE-cadherin	Santa Cruz, USA, sc-6458	Fig 2A, Immunofluorescence
pY658-VE-cadherin	Invitrogen, USA, #44-1144G	Fig 2B, immunoblot
pMLC	Cell Signaling Technology, USA, #3674	Fig 2B, immunoblot
Actin	Abcam, USA, mAbcam 8226	Fig 2B, immunoblot
Goat anti-rabbit IgG, HRP	BioActs, Korea, RSA1221	immunoblot
Goat anti-mouse IgG, HRP	BioActs, Korea, RSA1122	immunoblot

pY658-VE-cadherin antibody was produced against a chemically synthesized phosphopeptide derived from the region of human VE-cadherin that contains tyrosine 658. This sequence is conserved in chicken, chimpanzee, cow, dog, mouse, pig, and rat. The epitope of Santa Cruz VE-cadherin antibody is located at the C-terminus of VE-cadherin of human origin.

### In vitro permeability assay

BAECs were replated on plates coated with biotinylated fibronectin and grown to a dense monolayer. After incubation with compounds for 90 min, LPS (2 ug/mL) was added to the cells for 3 h. The cells were washed twice with phosphate-buffered saline (PBS) and incubated with fluorescein isothiocyanate (FITC)-streptavidin (Invitrogen) for 3 min, washed with PBS and fixed with formaldehyde. The nuclei of the fixed cells were washed, stained, and imaged under fluorescence microscopy. The area with fluorescence was measured after color thresholding using Image J(NIH). Transwell permeability assay was performed as previously reported [14] using Transwell Permeability Supports (Costar, 0.4 mM pore).

### Immunofluorescence staining

BAEC monolayers were stained with VE-cadherin antibody (Cell Signaling Technology 2158) and goat anti-rabbit IgG-FSD594 (BioActs, Korea). The slides were analyzed by fluorescence microscopy (Zeiss Axio Observer Z1).

### Animals

Mice were kept in a UNIST SPF animal facility, and the experiments were performed according to the animal protocol approved by UNIST Institutional Animal Care and Use Committee (Approval number: UNISTIACUC-22-32). Adult C57BL/6J male mice (6–8 weeks old, 20 – 30g) were used for *in vivo* analyses. After required treatment for experiments described below, the mice were euthanized using CO_2_ inhalation.

### LPS mouse model

After an intraperitoneal (IP) injection of mexenone, the mice were intravenously injected with LPS (18 mg/kg) and euthanized for analysis after 18 h (n = 6–13). Bronchial lavage (BAL) fluid was collected by the instillation of 1 mL of PBS, and the total cell numbers were determined using a LUNA Automated Cell Counter.

### Lung permeability measurement

Seventeen and a half hours after LPS injection (n = 7–11), mice tail veins were injected with Evans blue dye (Sigma-Aldrich; 2% solution in PBS, 4 mg/kg) and perfused after 30 min. The lungs were isolated, and the dye was extracted with formamide overnight at 50°C. The concentration of Evans blue was quantified by measuring absorbance at 611 nm and normalized to the dry weight of the lungs.

#### Histology analysis

After BAL fluid collection, lung tissue was isolated, and hematoxylin and eosin (H&E) staining was performed on 4 μm lung paraffin sections prepared by fixation in 10% neutral-buffered formalin and dehydration in 70% to 100% ethanol. Lung injury scoring was done as previously reported [15] and included (A) the number of neutrophils in the alveolar space, (B) interstitial space, and (C) hyaline membranes, (D) and proteinaceous debris in the airspace, but not alveolar septal thickening. The final lung injury score was calculated using the following formula: score  =  [(20 × A) + (14 × B) + (7 × C) + (7 × D)] / (number of fields × 100).

### Statistical analysis

Statistical analyses were performed using 2-way ANOVA or 1-way analysis of variance (ANOVA; multiple comparisons) in GraphPad Prism 6. Statistical significance was taken as p < 0.05. The data are presented as means ± SEM.

### Ethics approval

This study was approved by UNIST Institutional Animal Care and Use Committee (Approval number: UNISTIACUC-22-32).

## Results

### Identification of mexenone as a vascular permeability blocker

**Fig 1:**
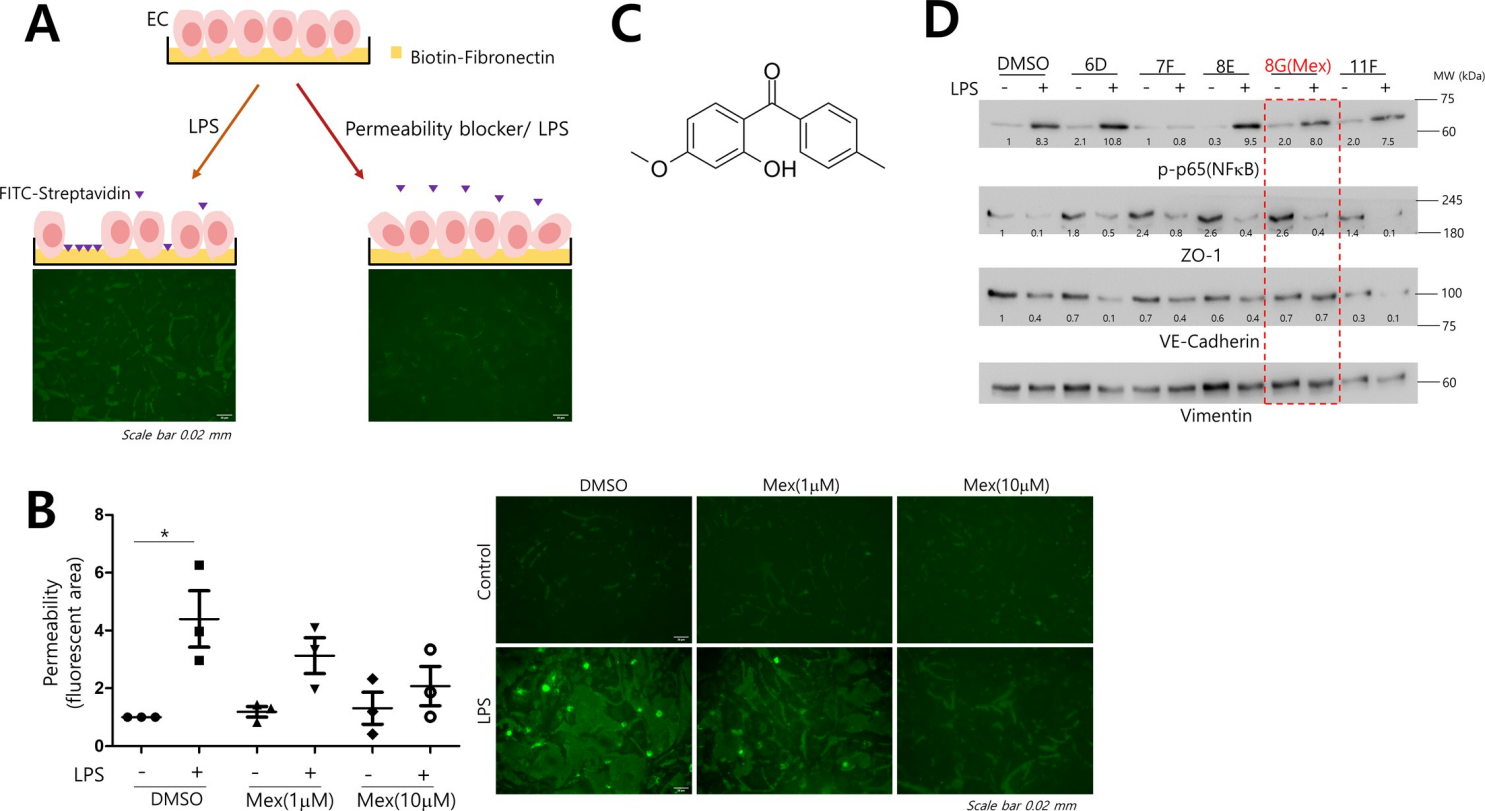
Identification of mexenone as a permeability blocker. A. BAECs were grown to a monolayer on biotin-labeled fibronectin for two days. After 90 min of drug (1 uM each) pretreatment, LPS (2 ug/mL) was added to the EC monolayer for 3 h to induce endothelial permeability. FITC-streptavidin was added and allowed to bind to exposed biotin-fibronectin for 3 min. After washing, the cells were fixed and observed by fluorescence microscopy. Fluorescent areas were imaged and quantified using ImageJ. B. Mexenone induced the blockade of endothelial permeability. BAEC monolayers were incubated with the indicated concentrations of mexenone for 90 min before stimulation with LPS for 3 h. The FITC-avidin bound area was calculated and normalized to the non-treated control group. *p < 0.05 (n = 3, two-way ANOVA,Bonferroni posttests). Permeability indicates the relative fold-increase of the FITC-stained area compared to non-treated controls. C. Chemical structure of mexenone (MW: 242.27), benzophenone-10, which has been used as a UV-absorbing agent in sunscreen cosmetics. D. BAEC monolayers were treated with clinical library compounds, including mexenone, stimulated with LPS for 3 h, and probed for the indicated antibodies by immunoblotting. BAEC:Bovine Endothelial Cells, LPS:Lipopolysaccharides.

For the *in vitro* endothelial permeability assay, an endothelial monolayer was formed on surfaces coated with biotinylated fibronectin (bio-FN). The confluent EC monolayer was starved for 6 h and treated with LPS for 3 h to induce endothelial permeability. LPS-induced passage and the binding of fluorescent avidin with bio-FN were measured by fluorescence microscopy (Fig 1A), and the stained areas were quantified using ImageJ (NIH). Among multiple hits from ∼3,000 compounds in the clinical drug library, mexenone significantly decreased LPS-induced permeability in a dose-dependent manner (Fig 1B). Mexenenone (benzophenone-10) is a benzophenone-derived UV filter used in sunscreen agents [16] (Fig 1C). We examined whether hits identified in screening targeted LPS-induced inflammatory signaling mediated by NFκB. Some hits suppressed NFκB activation, but mexenone blocked LPS-induced permeability without affecting NFκB activation (Fig 1D). Mexenone also blocked LPS-induced permeability in Human Lung Microvascular Endothelial Cells (HLMVECs) (S1 Fig).

### Modulation of VE-cadherin phosphorylation by mexenone

**Fig 2:**
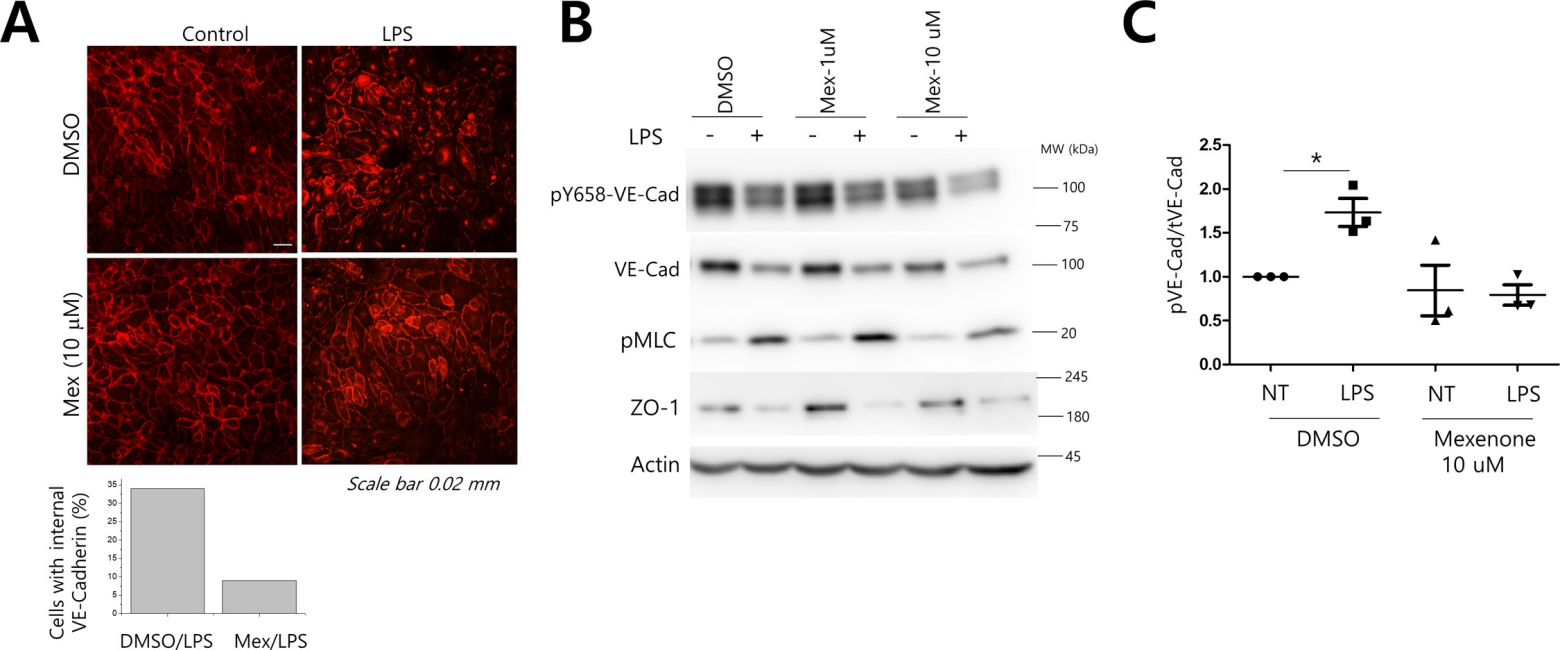
Effect of mexenone on VE-cadherin phosphorylation and localization. A. ECs were incubated with LPS after mexenone pretreatment and fixed and stained for VE-cadherin. B. Confluent BAECs were stimulated with LPS for 3 h with or without mexenone pretreatment. Cell lysates were prepared and subjected to immunoblotting with the indicated antibodies. C. The phosphorylation of VE-cadherin Y658 was measured using phospho-specific antibodies and normalized to total VE-cadherin expression (n = 3, two-way ANOVA, Bonferroni posttests, *p < 0.05). EC:Endothelial Cells.

EC barrier function is mainly mediated by two types of cell-cell adhesion. Adherens junctions (AJs) are EC-specific cell-cell junctions with VE-cadherin complexes. TJs are more ubiquitous and located apically to adherens junctions and contain multiple transmembrane proteins. To confirm the screening results, VE-cadherin was stained on endothelial monolayers after LPS treatment. LPS treatment led to weak junctional VE-cadherin and prominent perinuclear or nuclear localization. However, more VE-cadherin was seen in the junctions after mexenone pretreatment compared to the DMSO controls (Fig 2A). VE-cadherin phosphorylation is known to destabilize adherens junctions. We tested whether mexenone affected LPS-induced VE-cadherin phosphorylation. Y658 of VE-cadherin is reported to be phosphorylated by Src and mediate the binding of p120, an important stabilizer of VE-cadherin in junctions [17]. Endotoxins such as LPS have been shown to lead to VE-cadherin degradation [18,19]. LPS treatment for 3 h induced the degradation of VE-cadherin but was not affected by mexenone treatment. Interestingly, the examination of Y658 phosphorylation normalized by total VE-cadherin levels showed an LPS-dependent increase in VE-cadherin Y658 phosphorylation, which was completely blocked by mexenone treatment (Fig 2B and 2C). Actin cytoskeletal contractility measured by myosin light-chain phosphorylation or TJ protein ZO-1 levels was not affected by mexenone treatment (Fig 2B). The results suggest that mexenone may affect adherens junction complexes to prevent VE-cadherin Y658 phosphorylation and, thereby, promote barrier function.

### Mexenone mediates the blockade of LPS-induced lung injury

Vascular permeability is the main cause of organ damage during sepsis. Acute lung injury (ALI) and acute respiratory distress syndrome (ARDS) due to sepsis cause alveolar damage from increased permeability of the alveolar-capillary membrane, neutrophil sequestration, and impaired gas exchange [20].

**Fig 3:**
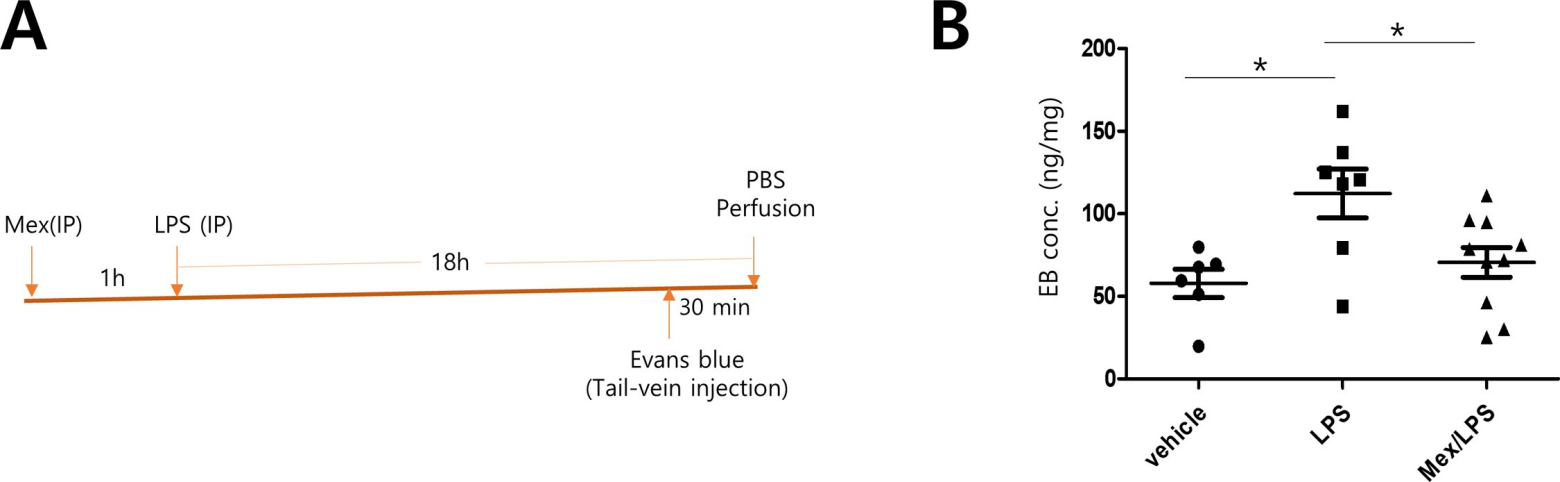
Effect of mexenone on LPS-induced lung permeability *in vivo*. A. *In vivo* permeability study design. Mexenone (10 mg/kg) was intraperitoneally injected into mice 1 h before LPS injection. Seventeen and a half hours after LPS injection, the mice were tail vein-injected with Evans blue dye (4 mg/kg) and perfused after 30 min of circulation. The lungs were isolated, and the dye was extracted and quantified. B. Evans blue dye was extracted from the lungs and quantified spectrophotometrically by measuring absorbance at 611 nm. One-way ANOVA, Tukey’s multiple comparison test, *p < 0.05.

We decided to test whether mexenone could prevent sepsis-induced lung injury in a mouse model. We first examined LPS-induced lung vascular permeability using the Evans blue dye extravasation assay (Fig 3A). LPS was injected 1 h after an IP injection of mexenone (10 mg/kg), then Evans blue dye was intravenously injected 30 min before PBS perfusion LPS significantly increased Evans blue dye leakage in lungs, but the venous injection of mexenone efficiently blocked the leakage (Fig 3B).

**Fig 4:**
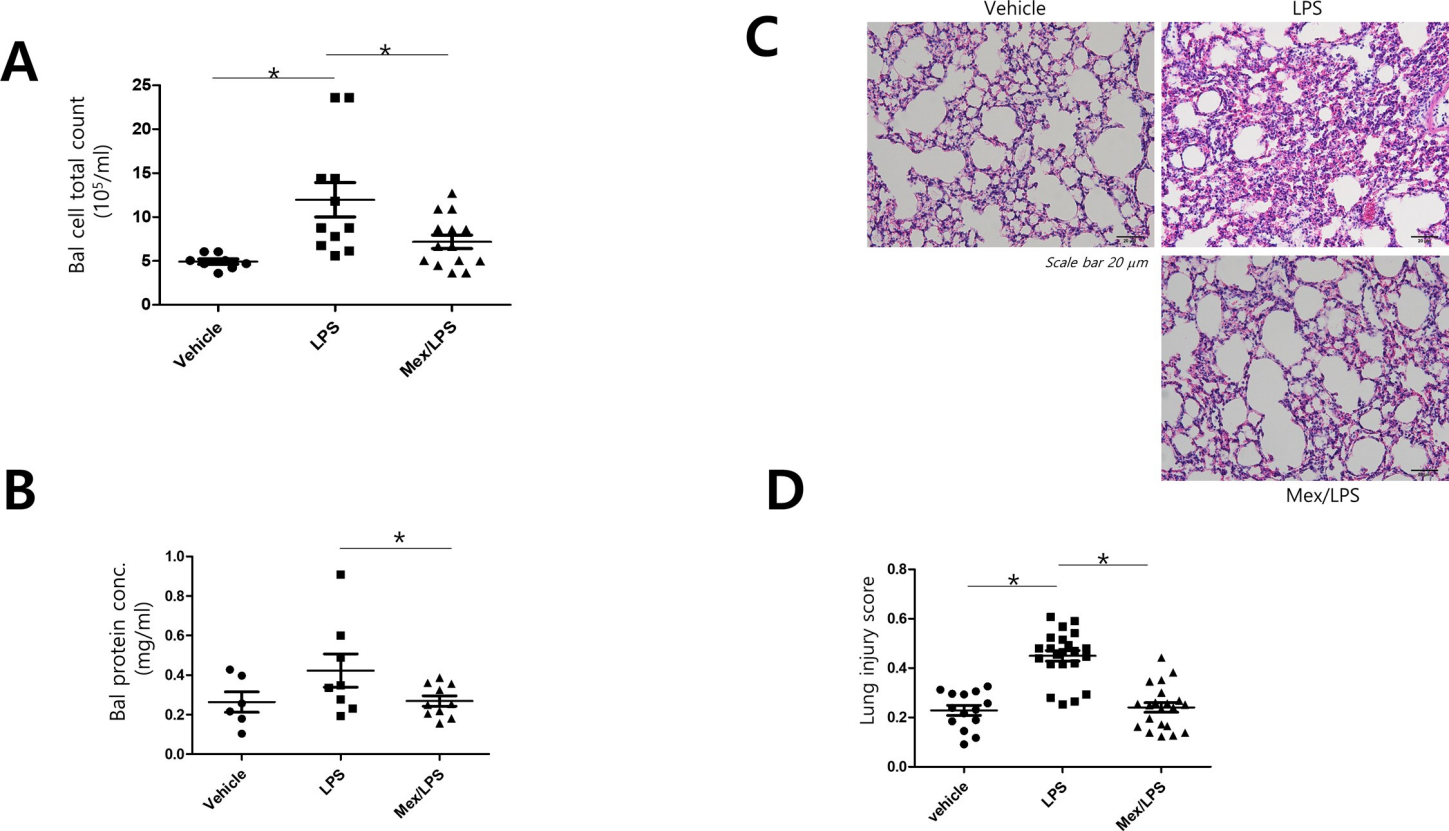
Mexenone prevents LPS-induced lung injury. A. Mice were intraperitoneally injected with vehicle or mexenone (10 mg/kg) for 1 h before the intraperitoneal injection of LPS (18 mg/kg). BAL fluid was collected by PBS instillation, and total cell numbers were counted. One-way ANOVA, Tukey’s multiple comparison test, *p < 0.05. B. Protein concentrations in BAL fluid were measured by the Bradford assay (n = 6–10, Student’s t-test). C. Lung tissue morphology changes were demonstrated by H&E staining of the control group, LPS group, and mexenone/LPS group. D. Histological lung injury scores were analyzed (n = 14–22, one-way ANOVA, Tukey’s multiple comparison test, *p < 0.05). BAL:Bronchoalveolar lavage.

We then prepared BAL fluid from LPS-injected mice with or without mexenone pretreatment. LPS injection increased cell counts and protein in BAL fluid, and the effects were blocked by mexenone injection (Figs 4A, 4B and S3A). Histopathological changes were examined by H&E staining. LPS-induced lung damages exhibited aggravated alveolar wall thickening, inflammatory cells, alveolar hemorrhage, hyaline membrane formation, and alveolar collapse compared to the control group. However, the mexenone-treated group showed significant alleviation of the pathological changes by LPS exposure (Figs 4C and S3B). The lung injury scores of the control, mexenone-treated, and LPS-treated groups supported these results (Fig 4D). In addition, post LPS administration of Mexenone protected mice from LPS-induced lung injury (S4 Fig). These results indicated that mexenone could mitigate LPS-induced lung injury from sepsis by blocking endothelial permeability.

## Discussion

Increased endothelial permeability and tissue edema in patients with sepsis have been hallmark features leading to fatal organ failure. The accumulation of fluids in tissues impairs organ function by suppressing oxygen diffusion and microvascular perfusion due to increased interstitial pressure [21]. Several reports showed that endothelial junction compromise was responsible for increased deaths in sepsis model animals [22,23]. Mexenone, identified in a cell-based screening for endothelial permeability blockers, effectively blocked vascular permeability and sepsis in a mouse model. The beneficial effect of mexenone was due to the specific blockade of LPS-induced VE-cadherin Y685 phosphorylation and endothelial barrier strengthening. LPS-induced pro-inflammatory signaling was not affected by mexenone treatment. Limitation of this study is the lack of another sepsis models such as cecal slurry or cecal ligation and puncture.

Drugs targeting inflammation during sepsis have been unsuccessful in clinical trials, presumably due to the immune function in pathogen clearance in later stages of sepsis and the heterogeneous nature of the disease. Our study showed that strengthening the endothelial barrier without affecting inflammatory signaling could be an appropriate strategy for preventing organ failure and reducing mortality when it is combined with current therapy. Mexenone has been widely used on human skin, and little toxicity has been reported [16]. It was well tolerated by the mice tested in this study. Detailed pharmacodynamic and toxicity analyses and mechanism of action studies are required, but it seems that mexenone or its derivatives have the potential to be promising drugs for sepsis treatment. Detailed molecular mechanism for Mexenone-induced barrier enhancement needs to be addressed. But, based on the specific effect of Mexenone on VE-Cadherin phosphorylation, Src family kinases known to mediate VE-Cadherin phosphorylation or tyrosine phosphatase such as SHP2 might be direct or indirect targets of Mexenone.

Several reports suggested that endothelial barrier function could be a therapeutic target for sepsis treatment. Robo ligand slit reduced sepsis mortality without affecting cytokine levels [22]. The activation of Tie2 using an agonistic antibody was effective in sepsis treatment, mainly via vascular stabilization [23]. However, these studies could not determine whether their beneficial effects on sepsis were from strengthening barrier function or other effects, including anti-inflammatory responses. Our study demonstrated that EC junction stabilization was sufficient for preventing ALI from sepsis by utilizing compounds from EC junction stabilization screening. To our knowledge, mexenone is the only small molecule whose anti-septic function was specifically derived from its effect on junction stability. Given the heterogeneous nature of the disease, targeting common vascular barrier disruption would be more effective for a broad range of sepsis cases.

Endothelial permeability plays an important role in other vascular diseases, including pulmonary hypertension [24], atherosclerosis [25], cancer metastasis [26], diabetic macular edema [27], and stroke [28]. Kwon et al. developed CU06-1004, a pseudo-sugar derivative of cholesterol, that blocked vascular permeability by preventing ligand-induced stress fiber formation and strengthening adherens junctions and TJs [29]. In a series of studies, this compound effectively ameliorated vascular permeability-related diseases, such as diabetic retinopathy [30], colitis [31], acute myocardial infarction [32], and hereditary angioedema [33]. Our results showing that mexenone reduced ALI by stabilizing the endothelial barrier also support the possibility that in many diseases, vascular permeability defects greatly contribute to disease outcomes, and the control of vascular permeability can be an effective therapeutic strategy.

Given the nature of mexenone’s specific targeting of VE-cadherin, the application of mexenone to other multiple vascular permeability diseases is expected to bring beneficial outcomes as well. The application of mexenone in other vascular inflammatory diseases is underway.

## Supporting information

S1 Raw images Unedited blot images. (PDF)

S1 Fig Mexenone prevents LPS-induced permeability in Human Lung Microvascular Endothelial Cells (HLMVECs). HLMVECs were grown to monolayers on fibronectin coated transwell inserts. After 90 min of Mexenone (10 μM) pretreatment, LPS (2 μg/mL) was added to the EC monolayer for 3 h to induce endothelial permeability. FITC-dextran (70kDa) was added to inner wells and outer well media was collected after 30 min and fluorescence was measured using plate reader (n = 3, one-way ANOVA, Tukey’s multiple comparison test, *p < 0.05).
(TIF)

S2 Fig Mexenone prevents high dose LPS-induced lung injury. A. Mice were intraperitoneally injected with vehicle or mexenone (10 mg/kg) for 1 h before the intraperitoneal injection of LPS (25 mg/kg). BAL fluid was collected by PBS instillation, and total cell numbers were counted (n = 4–7, one-way ANOVA, Tukey’s multiple comparison test, *p < 0.05). B. Lung tissue morphology changes were demonstrated by H&E staining and histological lung injury scores were analyzed (n = 10–20 fields, one-way ANOVA, Tukey’s multiple comparison test, *p < 0.05).
(TIF)

S3 Fig Post LPS administration of Mexenone protects from LPS-induced lung injury. Mice were intraperitoneally injected with vehicle or mexenone (10 mg/kg) for 2 h after the intraperitoneal injection of LPS (18 mg/kg). BAL cell counts (A, n = 4, one-way ANOVA, Tukey’s multiple comparison test, *p < 0.05) and lung injury score (B, n = 9–15, one-way ANOVA, Tukey’s multiple comparison test, *p < 0.05) were measured as in S1 Fig.
(TIF)

S4 Fig Morphological features used for lung injury scores. Arrows and circles indicate pathological morphological features.
(TIF)

S1 Data set Excel file containing all the data set used in the manuscript. (XLSX)
