# Supplementary figures and images for: Mexenone protects mice from LPS-induced sepsis by EC barrier stabilization
Source: PLoS One. 2024 May 9;19(5):e0302628. doi: 10.1371/journal.pone.0302628 (PMC11081322; doi:10.1371/journal.pone.0302628)

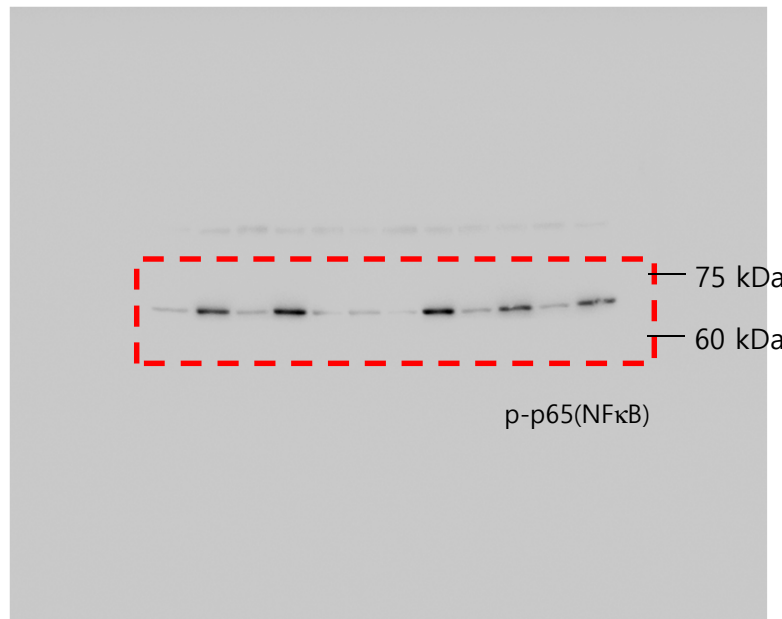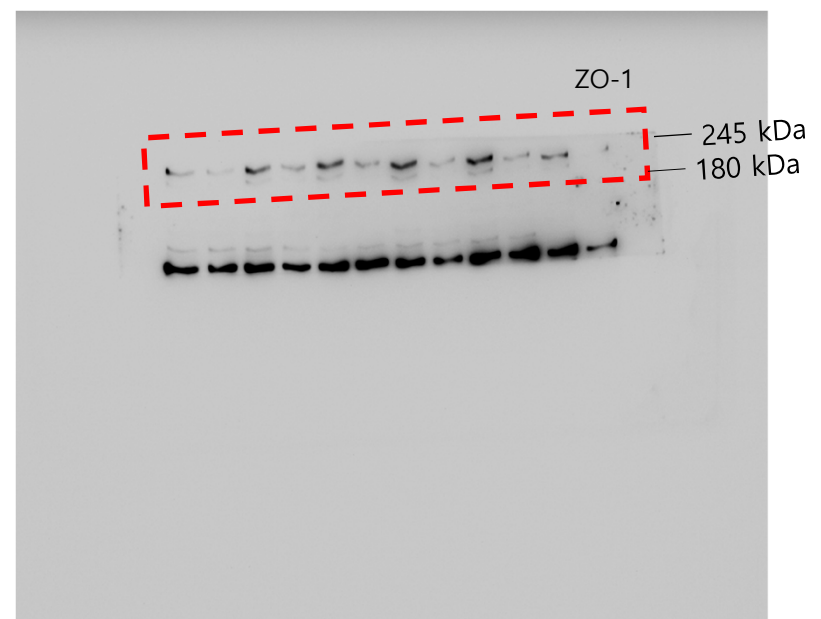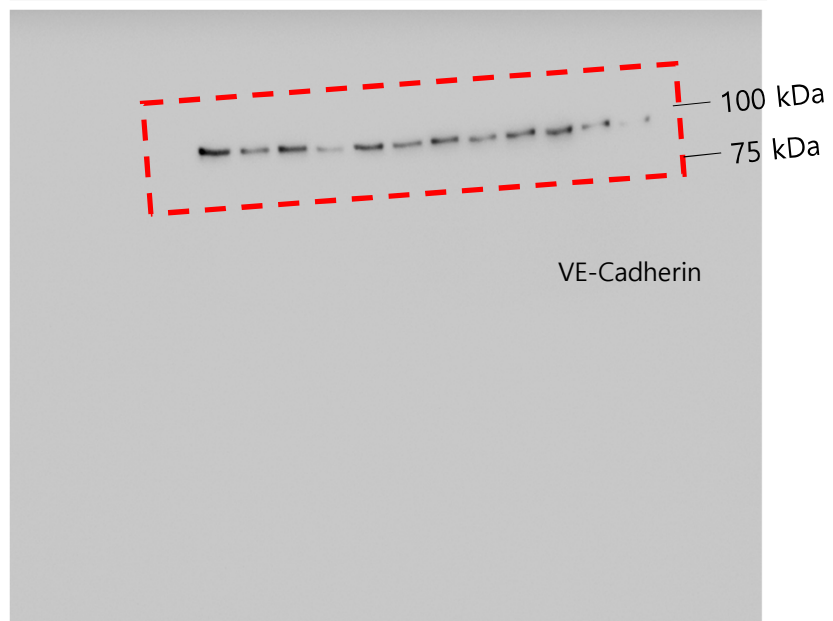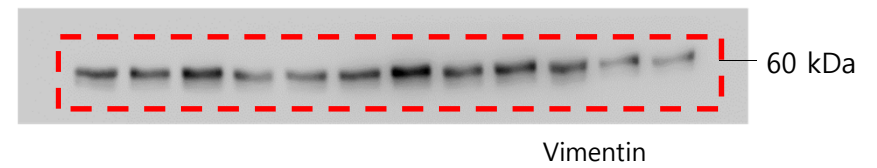

Figure 1D. Unedited blots

Figure 2B. Unedited blots

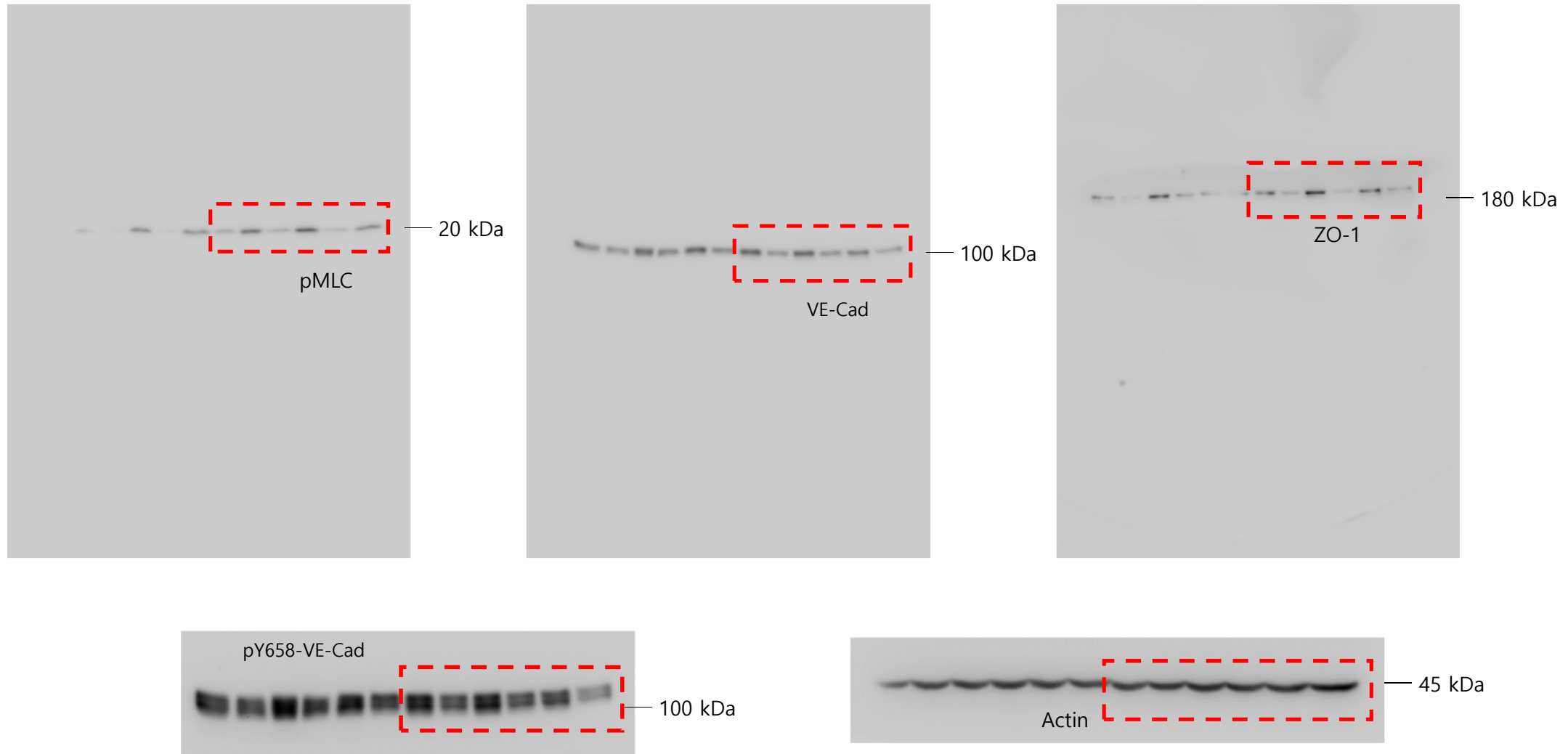

Supplement: S1 Raw images — (PDF) [file pone.0302628.s001.pdf]

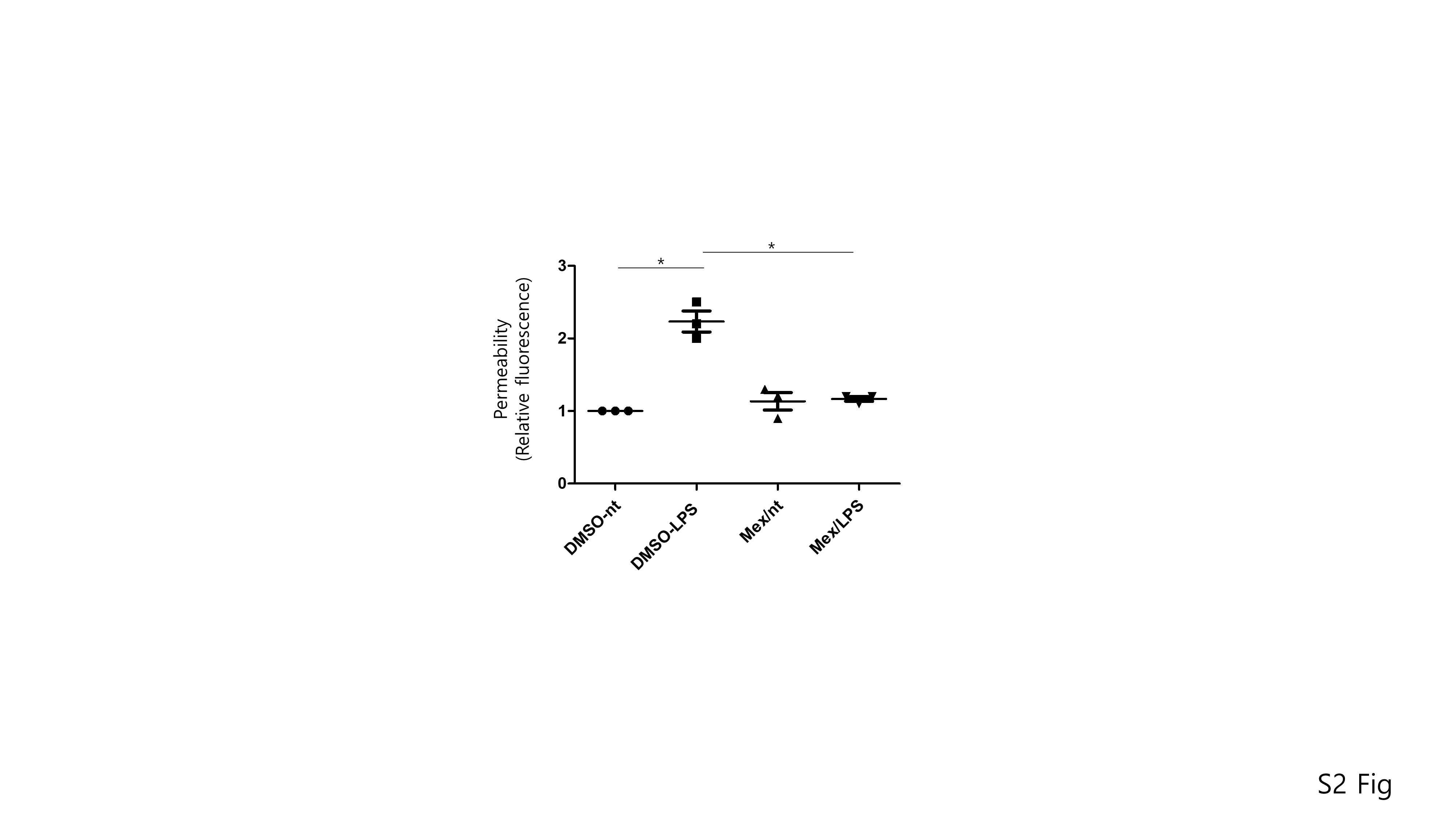

Supplement: S1 Fig — HLMVECs were grown to monolayers on fibronectin coated transwell inserts. After 90 min of Mexenone (10 μM) pretreatment, LPS (2 μg/mL) was added to the EC monolayer for 3 h to induce endothelial permeability. FITC-dextran (70kDa) was added to inner wells and outer well media was collected after 30 min and fluorescence was measured using plate reader (n = 3, one-way ANOVA, Tukey’s multiple comparison test, *p < 0.05). (TIF) [file pone.0302628.s002.tif]

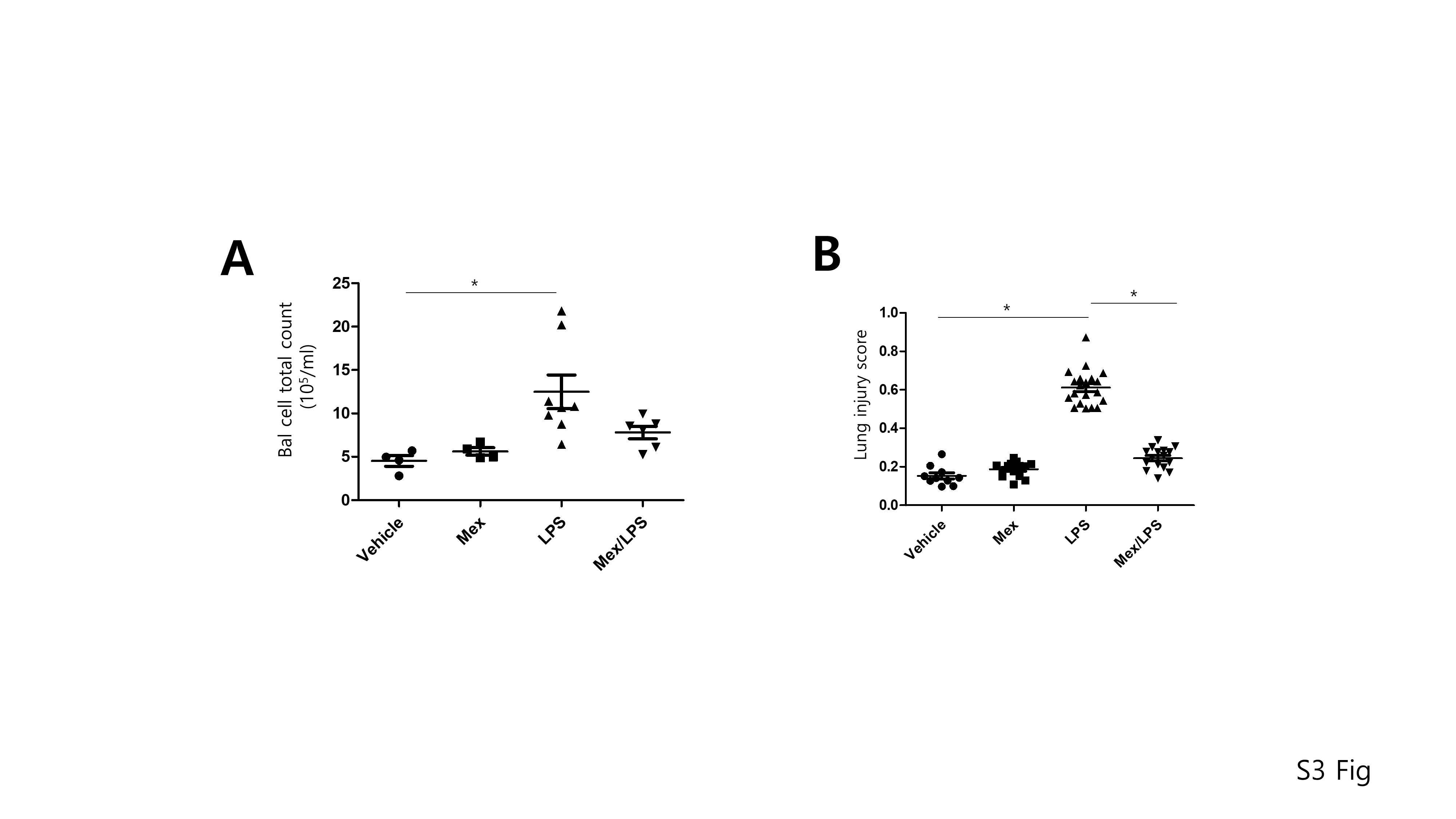

Supplement: S2 Fig — A. Mice were intraperitoneally injected with vehicle or mexenone (10 mg/kg) for 1 h before the intraperitoneal injection of LPS (25 mg/kg). BAL fluid was collected by PBS instillation, and total cell numbers were counted (n = 4–7, one-way ANOVA, Tukey’s multiple comparison test, *p < 0.05). B. Lung tissue morphology changes were demonstrated by H&E staining and histological lung injury scores were analyzed (n = 10–20 fields, one-way ANOVA, Tukey’s multiple comparison test, *p < 0.05). (TIF) [file pone.0302628.s003.tif]

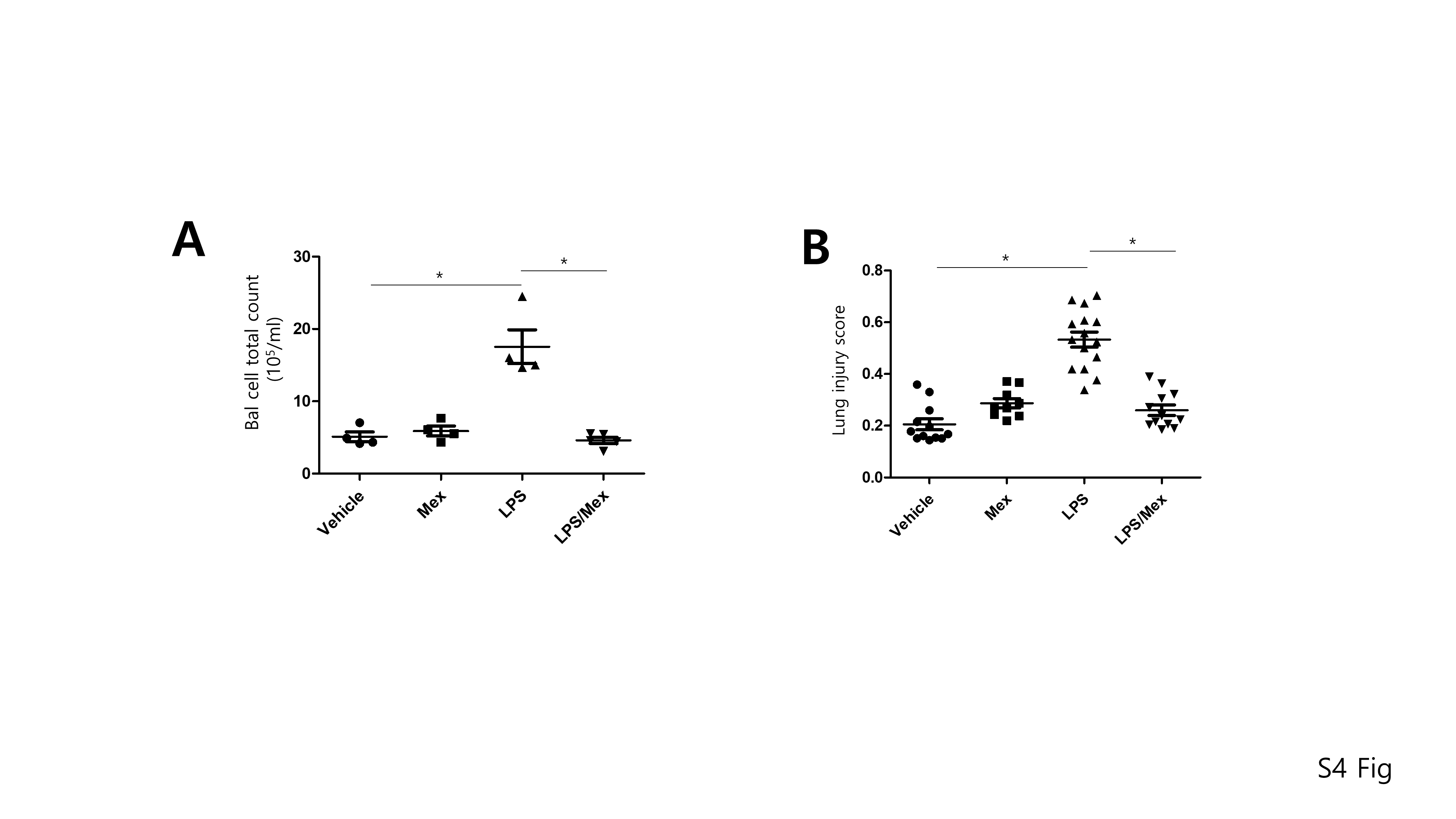

Supplement: S3 Fig — Mice were intraperitoneally injected with vehicle or mexenone (10 mg/kg) for 2 h after the intraperitoneal injection of LPS (18 mg/kg). BAL cell counts (A, n = 4, one-way ANOVA, Tukey’s multiple comparison test, *p < 0.05) and lung injury score (B, n = 9–15, one-way ANOVA, Tukey’s multiple comparison test, *p < 0.05) were measured as in S1 Fig. (TIF) [file pone.0302628.s004.tif]

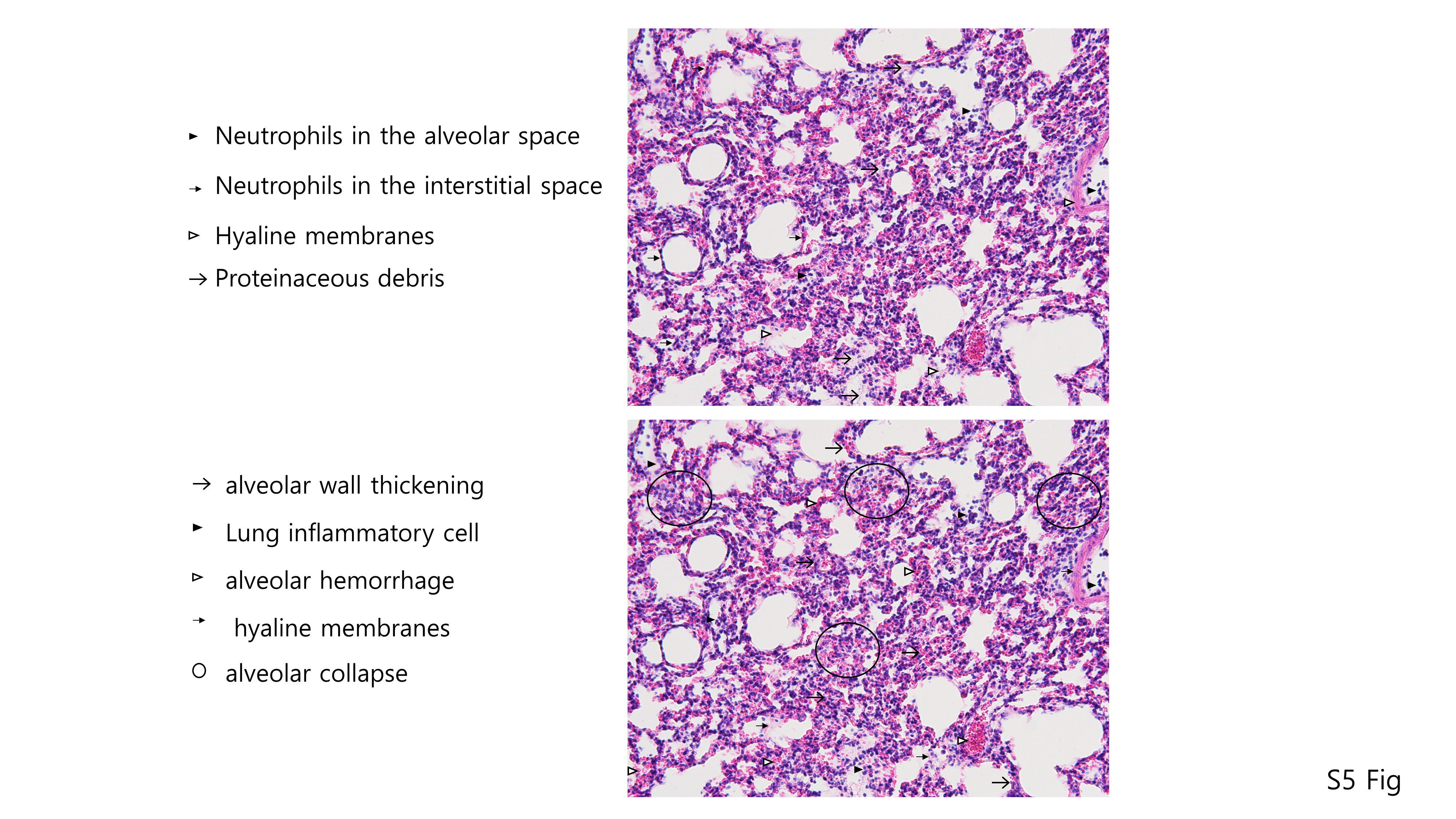

Supplement: S4 Fig — Arrows and circles indicate pathological morphological features. (TIF) [file pone.0302628.s005.tif]
