# Supplementary material for: Mexenone protects mice from LPS-induced sepsis by EC barrier stabilization
Source: PLoS One. 2024 May 9;19(5):e0302628. doi: 10.1371/journal.pone.0302628 (PMC11081322; doi:10.1371/journal.pone.0302628)
Supplement: Attachment — Submitted filename: point by point responses.docx [file pone.0302628.s007.docx]

Reviewer #1: Summary

The authors identify Mexenone from a compound library that reduces LPS-induced permeability in vitro. They also show that this compound can reduce LPS-induced permeability in mice. The study is well written and intriguing, but my concerns are below:

Major

1. It is unclear why aortic instead of lung endothelial cells were used for the in vitro screen. At the very least, the effect of Mexanone on LPS-induced permeability should be confirmed in lung endothelial cells in vitro.

Thank you for the suggestion. We’ve used Human Lung Microvascular Endothelial cells (HLMVECs) and confirmed that Mexenone prevents LPS-induced dextran-permeability in transwell permeability assay. The data is included in Supplementary Figure 1.

2. Thrombin-induced permeability as measured by trans-endothelial resistance is an established method to measure cell permeability in vitro. Regarding the method described in lines 68-74, is this an established method of assessing cell permeability in vitro? If so, references should be given to the papers that validated this method. If not, a more commonly used and established method should be used to confirm the effect of Mexanone in vitro.

As a more popular permeability assay, we’ve used transwell permeability assay measuring passage of FITC-dextran. The data is presented in Supplementary Figure 1.

3. The in vitro studies were prevention studies because the drug was given before LPS. Data could be included to assess the impact of adding the drug after LPS in vitro. Similarly, the in vivo studies are prevention studies because the mice received the drug before LPS. Data could be included to assess the impact of adding the drug after LPS in vivo. In the absence of additional data, this important issue should be mentioned in a Study Limitations section of the Discussion, and sentences regarding sepsis treatment should be avoided (e.g., line 172). Line 143 states the drug was given 18hrs before LPS. Fig 3A shows 1 hour. The timepoint of administration is not given in line 86. Please correct.

We’ve performed a new in vivo LPS experiment by injecting Mexenone two hours after LPS administration. Mexenone still effectively blocked LPS-induced lung injury and BAL fluid cell counts (Supplementary Figure 3). In line 143, 18h has been changed into 1h.

4. A second injury time point should be included for lung analyses (as well as 18h post-LPS).

Instead of checking longer injury time, we’ve added higher dose LPA treatment to strengthen our in vivo data (Supplementary Fig. 2).

5. Figs 1D and 2A should be quantified.

Figure 1D and 2A have been quantified

Minor

6. In the abstract, the cells used for the in vitro screening assay should be named. Also, in the abstract, the time points used for in vivo mexenone injections, and the time points of lung analyses should be included with respect to the time of LPS injection.

Corrected as suggested.

7. The potential mechanism(s) should be discussed.

A potential mechanism has been suggested in Discussion.

8. The limitations of the study should be discussed, including the absence of a second model in vitro or in vivo (e.g., cecal slurry or cecal ligation and puncture).

Limitation has been written in Discussion.

9. A more recent reference for the burden of sepsis could be included (line 23).

Another reference was added.

10. The sentences in lines 30-32 and 36-37 and 169-170 should be referenced.

References have been added.

11. In the abstract, the description of the drug library states 3000 drugs. In line 57, it states 2910 drugs. Please correct.

Abstract has been corrected.

12. Although the statistical analysis section refers to t tests, I do not see any of these tests included in the figure legends. Please correct. Also, Figures 1 and 2 should be analyzed by two-way ANOVA not one-way ANOVA, because there are two factors included (i.e., drug vs veh and LPS or no LPS).

Statistical analyses have been corrected in Methods section. Two-way ANOVA has been performed for Figure 1 and 2.

All graphs should include individual data points.

Individual data points were presented in all the plots.

Why are there so many data points on 4A Mex/LPS? It is stated n=6-10 in the methods.

Method has been corrected.

Reviewer #2: The current manuscript （PONE-D-23-14333）screened compound library in vitro to identify that mexenone can reduce LPS-induced endothelial cell leakage and inhibit LPS-induced lung injury in vivo. The mechanism may be related to the effect of mexenone on VE-Cad expression and phosphorylation. This research has certain value. Specific concerns are as follows:

1. The current research is to administer the drug in advance and then give LPS, which belongs to the category of prevention. If LPS is used to induce lung injury first, and then administered for treatment, can mexenone inhibit LPS-induced lung injury? This experiment should be supplemented.

Thank you for the important suggestion. We’ve performed a new in vivo LPS experiment by injecting Mexenone two hours after LPS administration. Mexenone still effectively blocked LPS-induced lung injury and BAL fluid cell counts (Supplementary Figure 3).

2. Does Mexenone have a protective effect on LPS-induced human endothelial cell injury? Please prove this experimentally.

We’ve used Human Lung Microvascular Endothelial cells (HLMVECs) and confirmed that Mexenone prevents LPS-induced dextran-permeability in transwell permeability assay. The data is included in Supplementary Figure 1.

3. Why is there many very bright spots in Fig. 1B? How to rule out endocytosis of the dye across the endothelial cell layer? How to determine that at the beginning, the junction of endothelial cells in each group has been formed and is the same? All figure legends should include information such as abbreviation definitions, number of trials, and statistics.

It’s not very clear but we assume the bright spots form where there are big holes between cells. We cannot entirely rule out the endocytosis of the dye but short (5 minute) dye incubation time would not be enough for transcytosis process. We started with same number of cells and there is no difference in culture condition before Mexenone or LPS treatment. Therefore, EC junction formation should be same in all wells. Figure legends have been amended.

4. In Fig.3A, nuclei should be stained with DAPI. Why are there two bands for phosphorylated VE-Cad but only one for total VE-Cad? Which one is the real target protein, please prove it. Why is VE-cad significantly reduced in a short period of time? What is the turnover cycle of VE-cad? All western blotting should be marked with a molecular marker, thereby indicating the size of the target protein.

Molecular makers have been added to immunoblot images. VE-Cadherin is susceptible to proteolysis and often leads to multiple bands. We assume epitope of phosphor-Y658 antibody exists in both uncleaved and cleaved VE-cadherin whereas VE-cad antibody detects only one band since epitope is located in the cleaved fragment. We don’t have nuclear staining for Figure 2A.

5. In the HE-stained images, the various pathological features described in Results should be marked with different arrows.

The pathological features have been noted with arrows in Supplementary Figure 4.

6. Some writing or typing need to be revised. For example, line58-59, 1 uM Compounds...etc.

Corrected.
