# Supplementary material for: Mexenone protects mice from LPS-induced sepsis by EC barrier stabilization
Source: PLoS One. 2024 May 9;19(5):e0302628. doi: 10.1371/journal.pone.0302628 (PMC11081322; doi:10.1371/journal.pone.0302628)
Supplement: Peer review history

# Peer review: Decision Letter 0

**DOI:** 10.1371/journal.pone.0302628.r001

**Authors:** You-Yang Zhao

3 Jul 2023

PONE-D-23-14333Mexenone protects mice from LPS-induced sepsis by EC barrier stabilizationPLOS ONE

Dear Dr. Yun,

Thank you for submitting your manuscript to PLOS ONE. After careful consideration, we feel that it has merit but does not fully meet PLOS ONE’s publication criteria as it currently stands. Therefore, we invite you to submit a revised version of the manuscript that addresses the points raised during the review process. There are several key experiments needed to be done, validation of human lung EC monolayer permeability with a common established method, e.g., FITC-dextran transwell assay with pre- and post-LPS treatment of the drug; Post-LPS treatment with the drug should be done in mice. Fig 3B shows mild injury induced by LPS. A more severe injury model with high er dose of LPS, i.p. should be tested.

==

Please submit your revised manuscript by Aug 17 2023 11:59PM. If you will need more time than this to complete your revisions, please reply to this message or contact the journal office at plosone@plos.org. When you're ready to submit your revision, log on to https://www.editorialmanager.com/pone/ and select the 'Submissions Needing Revision' folder to locate your manuscript file.

Please include the following items when submitting your revised manuscript:

- A rebuttal letter that responds to each point raised by the academic editor and reviewer(s). You should upload this letter as a separate file labeled 'Response to Reviewers'.
- A marked-up copy of your manuscript that highlights changes made to the original version. You should upload this as a separate file labeled 'Revised Manuscript with Track Changes'.
- An unmarked version of your revised paper without tracked changes. You should upload this as a separate file labeled 'Manuscript'.

If you would like to make changes to your financial disclosure, please include your updated statement in your cover letter. Guidelines for resubmitting your figure files are available below the reviewer comments at the end of this letter.

If applicable, we recommend that you deposit your laboratory protocols in protocols.io to enhance the reproducibility of your results. Protocols.io assigns your protocol its own identifier (DOI) so that it can be cited independently in the future. For instructions see: https://journals.plos.org/plosone/s/submission-guidelines#loc-laboratory-protocols. Additionally, PLOS ONE offers an option for publishing peer-reviewed Lab Protocol articles, which describe protocols hosted on protocols.io. Read more information on sharing protocols at https://plos.org/protocols?utm_medium=editorial-email&utm_source=authorletters&utm_campaign=protocols.

We look forward to receiving your revised manuscript.

Kind regards,

You-Yang Zhao

Academic Editor

PLOS ONE

Journal requirements:

When submitting your revision, we need you to address these additional requirements.

1. Please ensure that your manuscript meets PLOS ONE's style requirements, including those for file naming. The PLOS ONE style templates can be found at

https://journals.plos.org/plosone/s/file?id=wjVg/PLOSOne_formatting_sample_main_body.pdf and

https://journals.plos.org/plosone/s/file?id=ba62/PLOSOne_formatting_sample_title_authors_affiliations.pdf

2. Thank you for stating the following in the Acknowledgments Section of your manuscript:

“This research was supported by the Basic Science Research Program through the National Research Foundation of Korea (NRF) funded by the Ministry of Education (202103890002). We thank UNIST Central Research Facilities (UCRF) for their support in using the equipment. The chemical library used in this study was kindly provided by the Korea Chemical Bank (www.chembank.org) of the Korea Research Institute of Chemical Technology.”

We note that you have provided funding information that is currently declared in your Funding Statement. However, funding information should not appear in the Acknowledgments section or other areas of your manuscript. We will only publish funding information present in the Funding Statement section of the online submission form.

Please remove any funding-related text from the manuscript and let us know how you would like to update your Funding Statement. Currently, your Funding Statement reads as follows:

“This research was supported by the Basic Science Research Program awarded to SY through the National Research Foundation of Korea (NRF) funded by the Ministry of Education (202103890002).  The funders had no role in study design, data collection and analysis, decision to publish, or preparation of the manuscript.”

Please include your amended statements within your cover letter; we will change the online submission form on your behalf.

3. PLOS ONE now requires that authors provide the original uncropped and unadjusted images underlying all blot or gel results reported in a submission’s figures or Supporting Information files. This policy and the journal’s other requirements for blot/gel reporting and figure preparation are described in detail at https://journals.plos.org/plosone/s/figures#loc-blot-and-gel-reporting-requirements and https://journals.plos.org/plosone/s/figures#loc-preparing-figures-from-image-files. When you submit your revised manuscript, please ensure that your figures adhere fully to these guidelines and provide the original underlying images for all blot or gel data reported in your submission. See the following link for instructions on providing the original image data: https://journals.plos.org/plosone/s/figures#loc-original-images-for-blots-and-gels.

In your cover letter, please note whether your blot/gel image data are in Supporting Information or posted at a public data repository, provide the repository URL if relevant, and provide specific details as to which raw blot/gel images, if any, are not available. Email us at plosone@plos.org if you have any questions.

4. In your Data Availability statement, you have not specified where the minimal data set underlying the results described in your manuscript can be found. PLOS defines a study's minimal data set as the underlying data used to reach the conclusions drawn in the manuscript and any additional data required to replicate the reported study findings in their entirety. All PLOS journals require that the minimal data set be made fully available. For more information about our data policy, please see http://journals.plos.org/plosone/s/data-availability.

Upon re-submitting your revised manuscript, please upload your study’s minimal underlying data set as either Supporting Information files or to a stable, public repository and include the relevant URLs, DOIs, or accession numbers within your revised cover letter. For a list of acceptable repositories, please see http://journals.plos.org/plosone/s/data-availability#loc-recommended-repositories. Any potentially identifying patient information must be fully anonymized.

Important: If there are ethical or legal restrictions to sharing your data publicly, please explain these restrictions in detail. Please see our guidelines for more information on what we consider unacceptable restrictions to publicly sharing data: http://journals.plos.org/plosone/s/data-availability#loc-unacceptable-data-access-restrictions. Note that it is not acceptable for the authors to be the sole named individuals responsible for ensuring data access.

We will update your Data Availability statement to reflect the information you provide in your cover letter.

[Note: HTML markup is below. Please do not edit.]

Reviewers' comments:

Reviewer's Responses to Questions

**Comments to the Author**

1. Is the manuscript technically sound, and do the data support the conclusions?

The manuscript must describe a technically sound piece of scientific research with data that supports the conclusions. Experiments must have been conducted rigorously, with appropriate controls, replication, and sample sizes. The conclusions must be drawn appropriately based on the data presented. 

Reviewer #1: Partly

Reviewer #2: Yes

**********

2. Has the statistical analysis been performed appropriately and rigorously? 

Reviewer #1: No

Reviewer #2: Yes

**********

3. Have the authors made all data underlying the findings in their manuscript fully available?

The PLOS Data policy requires authors to make all data underlying the findings described in their manuscript fully available without restriction, with rare exception (please refer to the Data Availability Statement in the manuscript PDF file). The data should be provided as part of the manuscript or its supporting information, or deposited to a public repository. For example, in addition to summary statistics, the data points behind means, medians and variance measures should be available. If there are restrictions on publicly sharing data—e.g. participant privacy or use of data from a third party—those must be specified.

Reviewer #1: No

Reviewer #2: Yes

**********

4. Is the manuscript presented in an intelligible fashion and written in standard English?

PLOS ONE does not copyedit accepted manuscripts, so the language in submitted articles must be clear, correct, and unambiguous. Any typographical or grammatical errors should be corrected at revision, so please note any specific errors here.

Reviewer #1: Yes

Reviewer #2: Yes

**********

5. Review Comments to the Author

Please use the space provided to explain your answers to the questions above. You may also include additional comments for the author, including concerns about dual publication, research ethics, or publication ethics. (Please upload your review as an attachment if it exceeds 20,000 characters)

Reviewer #1: Summary

The authors identify Mexenone from a compound library that reduces LPS-induced permeability in vitro. They also show that this compound can reduce LPS-induced permeability in mice. The study is well written and intriguing, but my concerns are below:

Major

1. It is unclear why aortic instead of lung endothelial cells were used for the in vitro screen. At the very least, the effect of Mexanone on LPS-induced permeability should be confirmed in lung endothelial cells in vitro.

2. Thrombin-induced permeability as measured by trans-endothelial resistance is an established method to measure cell permeability in vitro. Regarding the method described in lines 68-74, is this an established method of assessing cell permeability in vitro? If so, references should be given to the papers that validated this method. If not, a more commonly used and established method should be used to confirm the effect of Mexanone in vitro.

3. The in vitro studies were prevention studies because the drug was given before LPS. Data could be included to assess the impact of adding the drug after LPS in vitro. Similarly, the in vivo studies are prevention studies because the mice received the drug before LPS. Data could be included to assess the impact of adding the drug after LPS in vivo. In the absence of additional data, this important issue should be mentioned in a Study Limitations section of the Discussion, and sentences regarding sepsis treatment should be avoided (e.g., line 172). Line 143 states the drug was given 18hrs before LPS. Fig 3A shows 1 hour. The timepoint of administration is not given in line 86. Please correct.

4. A second injury time point should be included for lung analyses (as well as 18h post-LPS).

5. Figs 1D and 2A should be quantified.

Minor

6. In the abstract, the cells used for the in vitro screening assay should be named. Also in the abstract, the time points used for in vivo mexenone injections, and the time points of lung analyses should be included with respect to the time of LPS injection.

7. The potential mechanism(s) should be discussed.

8. The limitations of the study should be discussed, including the absence of a second model in vitro or in vivo (e.g., cecal slurry or cecal ligation and puncture).

9. A more recent reference for the burden of sepsis could be included (line 23).

10. The sentences in lines 30-32 and 36-37 and 169-170 should be referenced.

11. In the abstract, the description of the drug library states 3000 drugs. In line 57, it states 2910 drugs. Please correct.

12. Although the statistical analysis section refers to t tests, I do not see any of these tests included in the figure legends. Please correct. Also, Figures 1 and 2 should be analyzed by two-way ANOVA not one-way ANOVA, because there are two factors included (i.e., drug vs veh and LPS or no LPS). All graphs should include individual data points. Why are there so many data points on 4A Mex/LPS? It is stated n=6-10 in the methods.

Reviewer #2: The current manuscript （PONE-D-23-14333）screened compound library in vitro to identify that mexenone can reduce LPS-induced endothelial cell leakage and inhibit LPS-induced lung injury in vivo. The mechanism may be related to the effect of mexenone on VE-Cad expression and phosphorylation. This research has certain value. Specific concerns are as follows:

1. The current research is to administer the drug in advance and then give LPS, which belongs to the category of prevention. If LPS is used to induce lung injury first, and then administered for treatment, can mexenone inhibit LPS-induced lung injury? This experiment should be supplemented.

2. Does Mexenone have a protective effect on LPS-induced human endothelial cell injury? Please prove this experimentally.

3. Why is there many very bright spots in Fig. 1B? How to rule out endocytosis of the dye across the endothelial cell layer? How to determine that at the beginning, the junction of endothelial cells in each group has been formed and is the same? All figure legends should include information such as abbreviation definitions, number of trials, and statistics.

4. In Fig.3A, nuclei should be stained with DAPI. Why are there two bands for phosphorylated VE-Cad but only one for total VE-Cad? Which one is the real target protein, please prove it. Why is VE-cad significantly reduced in a short period of time? What is the turnover cycle of VE-cad? All western blotting should be marked with a molecular marker, thereby indicating the size of the target protein.

5. In the HE-stained images, the various pathological features described in Results should be marked with different arrows.

6. Some writing or typing need to be revised. For example, line58-59, 1 uM Compounds...etc.

**********

6. PLOS authors have the option to publish the peer review history of their article (what does this mean?). If published, this will include your full peer review and any attached files.

If you choose “no”, your identity will remain anonymous but your review may still be made public.

**Do you want your identity to be public for this peer review?** For information about this choice, including consent withdrawal, please see our Privacy Policy.

Reviewer #1: **Yes: **Colin Evans

Reviewer #2: No

**********

[NOTE: If reviewer comments were submitted as an attachment file, they will be attached to this email and accessible via the submission site. Please log into your account, locate the manuscript record, and check for the action link "View Attachments". If this link does not appear, there are no attachment files.]

While revising your submission, please upload your figure files to the Preflight Analysis and Conversion Engine (PACE) digital diagnostic tool, https://pacev2.apexcovantage.com/. PACE helps ensure that figures meet PLOS requirements. To use PACE, you must first register as a user. Registration is free. Then, login and navigate to the UPLOAD tab, where you will find detailed instructions on how to use the tool. If you encounter any issues or have any questions when using PACE, please email PLOS at figures@plos.org. Please note that Supporting Information files do not need this step.

# Author comment: Author response to Decision Letter 0

**DOI:** 10.1371/journal.pone.0302628.r002

21 Jan 2024

Reviewer #1: Summary

The authors identify Mexenone from a compound library that reduces LPS-induced permeability in vitro. They also show that this compound can reduce LPS-induced permeability in mice. The study is well written and intriguing, but my concerns are below:

Major

1. It is unclear why aortic instead of lung endothelial cells were used for the in vitro screen. At the very least, the effect of Mexanone on LPS-induced permeability should be confirmed in lung endothelial cells in vitro.

Thank you for the suggestion. We’ve used Human Lung Microvascular Endothelial cells (HLMVECs) and confirmed that Mexenone prevents LPS-induced dextran-permeability in transwell permeability assay. The data is included in Supplementary Figure 1. 

2. Thrombin-induced permeability as measured by trans-endothelial resistance is an established method to measure cell permeability in vitro. Regarding the method described in lines 68-74, is this an established method of assessing cell permeability in vitro? If so, references should be given to the papers that validated this method. If not, a more commonly used and established method should be used to confirm the effect of Mexanone in vitro.

As a more popular permeability assay, we’ve used transwell permeability assay measuring passage of FITC-dextran. The data is presented in Supplementary Figure 1. 

3. The in vitro studies were prevention studies because the drug was given before LPS. Data could be included to assess the impact of adding the drug after LPS in vitro. Similarly, the in vivo studies are prevention studies because the mice received the drug before LPS. Data could be included to assess the impact of adding the drug after LPS in vivo. In the absence of additional data, this important issue should be mentioned in a Study Limitations section of the Discussion, and sentences regarding sepsis treatment should be avoided (e.g., line 172). Line 143 states the drug was given 18hrs before LPS. Fig 3A shows 1 hour. The timepoint of administration is not given in line 86. Please correct.

We’ve performed a new in vivo LPS experiment by injecting Mexenone two hours after LPS administration. Mexenone still effectively blocked LPS-induced lung injury and BAL fluid cell counts (Supplementary Figure 3). In line 143, 18h has been changed into 1h. 

4. A second injury time point should be included for lung analyses (as well as 18h post-LPS).

Instead of checking longer injury time, we’ve added higher dose LPA treatment to strengthen our in vivo data (Supplementary Fig. 2). 

5. Figs 1D and 2A should be quantified.

Figure 1D and 2A have been quantified

Minor

6. In the abstract, the cells used for the in vitro screening assay should be named. Also, in the abstract, the time points used for in vivo mexenone injections, and the time points of lung analyses should be included with respect to the time of LPS injection.

Corrected as suggested.

7. The potential mechanism(s) should be discussed.

A potential mechanism has been suggested in Discussion.

8. The limitations of the study should be discussed, including the absence of a second model in vitro or in vivo (e.g., cecal slurry or cecal ligation and puncture).

Limitation has been written in Discussion.

9. A more recent reference for the burden of sepsis could be included (line 23).

Another reference was added. 

10. The sentences in lines 30-32 and 36-37 and 169-170 should be referenced.

References have been added.

11. In the abstract, the description of the drug library states 3000 drugs. In line 57, it states 2910 drugs. Please correct.

Abstract has been corrected.

12. Although the statistical analysis section refers to t tests, I do not see any of these tests included in the figure legends. Please correct. Also, Figures 1 and 2 should be analyzed by two-way ANOVA not one-way ANOVA, because there are two factors included (i.e., drug vs veh and LPS or no LPS). 

Statistical analyses have been corrected in Methods section. Two-way ANOVA has been performed for Figure 1 and 2. 

All graphs should include individual data points. 

Individual data points were presented in all the plots. 

Why are there so many data points on 4A Mex/LPS? It is stated n=6-10 in the methods.

Method has been corrected.

Reviewer #2: The current manuscript （PONE-D-23-14333）screened compound library in vitro to identify that mexenone can reduce LPS-induced endothelial cell leakage and inhibit LPS-induced lung injury in vivo. The mechanism may be related to the effect of mexenone on VE-Cad expression and phosphorylation. This research has certain value. Specific concerns are as follows:

1. The current research is to administer the drug in advance and then give LPS, which belongs to the category of prevention. If LPS is used to induce lung injury first, and then administered for treatment, can mexenone inhibit LPS-induced lung injury? This experiment should be supplemented.

Thank you for the important suggestion. We’ve performed a new in vivo LPS experiment by injecting Mexenone two hours after LPS administration. Mexenone still effectively blocked LPS-induced lung injury and BAL fluid cell counts (Supplementary Figure 3). 

2. Does Mexenone have a protective effect on LPS-induced human endothelial cell injury? Please prove this experimentally.

We’ve used Human Lung Microvascular Endothelial cells (HLMVECs) and confirmed that Mexenone prevents LPS-induced dextran-permeability in transwell permeability assay. The data is included in Supplementary Figure 1. 

3. Why is there many very bright spots in Fig. 1B? How to rule out endocytosis of the dye across the endothelial cell layer? How to determine that at the beginning, the junction of endothelial cells in each group has been formed and is the same? All figure legends should include information such as abbreviation definitions, number of trials, and statistics.

It’s not very clear but we assume the bright spots form where there are big holes between cells. We cannot entirely rule out the endocytosis of the dye but short (5 minute) dye incubation time would not be enough for transcytosis process. We started with same number of cells and there is no difference in culture condition before Mexenone or LPS treatment. Therefore, EC junction formation should be same in all wells. Figure legends have been amended. 

4. In Fig.3A, nuclei should be stained with DAPI. Why are there two bands for phosphorylated VE-Cad but only one for total VE-Cad? Which one is the real target protein, please prove it. Why is VE-cad significantly reduced in a short period of time? What is the turnover cycle of VE-cad? All western blotting should be marked with a molecular marker, thereby indicating the size of the target protein.

Molecular makers have been added to immunoblot images. VE-Cadherin is susceptible to proteolysis and often leads to multiple bands. We assume epitope of phosphor-Y658 antibody exists in both uncleaved and cleaved VE-cadherin whereas VE-cad antibody detects only one band since epitope is located in the cleaved fragment. We don’t have nuclear staining for Figure 2A. 

5. In the HE-stained images, the various pathological features described in Results should be marked with different arrows.

The pathological features have been noted with arrows in Supplementary Figure 4. 

6. Some writing or typing need to be revised. For example, line58-59, 1 uM Compounds...etc.

Corrected.

Attachment Submitted filename: point by point responses.docx

# Peer review: Decision Letter 1

**DOI:** 10.1371/journal.pone.0302628.r003

**Authors:** You-Yang Zhao

15 Mar 2024

PONE-D-23-14333R1Mexenone protects mice from LPS-induced sepsis by EC barrier stabilizationPLOS ONE

Dear Dr. Yun,

Thank you for submitting your manuscript to PLOS ONE. After careful consideration, we feel that it has merit but does not fully meet PLOS ONE’s publication criteria as it currently stands. Therefore, we invite you to submit a revised version of the manuscript that addresses the points raised during the review process. For VE-Cad Western blotting, please cite the relevant literature showing that VE-cad is easy to be degraded and has shown Western blotting results similar to the manuscript. In addition, the epitopes of the anti-VE-cad and anti-phosphorylated VE-cad antibodies should be provided in the method.

Please submit your revised manuscript by Apr 29 2024 11:59PM. If you will need more time than this to complete your revisions, please reply to this message or contact the journal office at plosone@plos.org. When you're ready to submit your revision, log on to https://www.editorialmanager.com/pone/ and select the 'Submissions Needing Revision' folder to locate your manuscript file.

Please include the following items when submitting your revised manuscript:

- A rebuttal letter that responds to each point raised by the academic editor and reviewer(s). You should upload this letter as a separate file labeled 'Response to Reviewers'.
- A marked-up copy of your manuscript that highlights changes made to the original version. You should upload this as a separate file labeled 'Revised Manuscript with Track Changes'.
- An unmarked version of your revised paper without tracked changes. You should upload this as a separate file labeled 'Manuscript'.

If you would like to make changes to your financial disclosure, please include your updated statement in your cover letter. Guidelines for resubmitting your figure files are available below the reviewer comments at the end of this letter.

If applicable, we recommend that you deposit your laboratory protocols in protocols.io to enhance the reproducibility of your results. Protocols.io assigns your protocol its own identifier (DOI) so that it can be cited independently in the future. For instructions see: https://journals.plos.org/plosone/s/submission-guidelines#loc-laboratory-protocols. Additionally, PLOS ONE offers an option for publishing peer-reviewed Lab Protocol articles, which describe protocols hosted on protocols.io. Read more information on sharing protocols at https://plos.org/protocols?utm_medium=editorial-email&utm_source=authorletters&utm_campaign=protocols.

We look forward to receiving your revised manuscript.

Kind regards,

You-Yang Zhao

Academic Editor

PLOS ONE

Journal Requirements:

Please review your reference list to ensure that it is complete and correct. If you have cited papers that have been retracted, please include the rationale for doing so in the manuscript text, or remove these references and replace them with relevant current references. Any changes to the reference list should be mentioned in the rebuttal letter that accompanies your revised manuscript. If you need to cite a retracted article, indicate the article’s retracted status in the References list and also include a citation and full reference for the retraction notice.

[Note: HTML markup is below. Please do not edit.]

Reviewers' comments:

Reviewer's Responses to Questions

**Comments to the Author**

1. If the authors have adequately addressed your comments raised in a previous round of review and you feel that this manuscript is now acceptable for publication, you may indicate that here to bypass the “Comments to the Author” section, enter your conflict of interest statement in the “Confidential to Editor” section, and submit your "Accept" recommendation.

Reviewer #3: All comments have been addressed

Reviewer #4: (No Response)

**********

2. Is the manuscript technically sound, and do the data support the conclusions?

The manuscript must describe a technically sound piece of scientific research with data that supports the conclusions. Experiments must have been conducted rigorously, with appropriate controls, replication, and sample sizes. The conclusions must be drawn appropriately based on the data presented. 

Reviewer #3: (No Response)

Reviewer #4: Yes

**********

3. Has the statistical analysis been performed appropriately and rigorously? 

Reviewer #3: (No Response)

Reviewer #4: Yes

**********

4. Have the authors made all data underlying the findings in their manuscript fully available?

The PLOS Data policy requires authors to make all data underlying the findings described in their manuscript fully available without restriction, with rare exception (please refer to the Data Availability Statement in the manuscript PDF file). The data should be provided as part of the manuscript or its supporting information, or deposited to a public repository. For example, in addition to summary statistics, the data points behind means, medians and variance measures should be available. If there are restrictions on publicly sharing data—e.g. participant privacy or use of data from a third party—those must be specified.

Reviewer #3: (No Response)

Reviewer #4: Yes

**********

5. Is the manuscript presented in an intelligible fashion and written in standard English?

PLOS ONE does not copyedit accepted manuscripts, so the language in submitted articles must be clear, correct, and unambiguous. Any typographical or grammatical errors should be corrected at revision, so please note any specific errors here.

Reviewer #3: (No Response)

Reviewer #4: Yes

**********

6. Review Comments to the Author

Please use the space provided to explain your answers to the questions above. You may also include additional comments for the author, including concerns about dual publication, research ethics, or publication ethics. (Please upload your review as an attachment if it exceeds 20,000 characters)

Reviewer #3: (No Response)

Reviewer #4: The revised manuscript answers most of the questions properly, but there is still a minor question about Western blotting for VE-cad. Please cite the relevant literature showing that Ve-cad is easy to degrade and has obtained Western blotting results similar to the manuscript. In addition, the recognition epitopes of the anti-VE-cad and anti-phosphorylated VE-cad antibodies used should be shown. 

**********

7. PLOS authors have the option to publish the peer review history of their article (what does this mean?). If published, this will include your full peer review and any attached files.

If you choose “no”, your identity will remain anonymous but your review may still be made public.

**Do you want your identity to be public for this peer review?** For information about this choice, including consent withdrawal, please see our Privacy Policy.

Reviewer #3: **Yes: **Colin Evans

Reviewer #4: No

**********

[NOTE: If reviewer comments were submitted as an attachment file, they will be attached to this email and accessible via the submission site. Please log into your account, locate the manuscript record, and check for the action link "View Attachments". If this link does not appear, there are no attachment files.]

While revising your submission, please upload your figure files to the Preflight Analysis and Conversion Engine (PACE) digital diagnostic tool, https://pacev2.apexcovantage.com/. PACE helps ensure that figures meet PLOS requirements. To use PACE, you must first register as a user. Registration is free. Then, login and navigate to the UPLOAD tab, where you will find detailed instructions on how to use the tool. If you encounter any issues or have any questions when using PACE, please email PLOS at figures@plos.org. Please note that Supporting Information files do not need this step.

# Author comment: Author response to Decision Letter 1

**DOI:** 10.1371/journal.pone.0302628.r004

18 Mar 2024

For VE-Cad Western blotting, please cite the relevant literature showing that VE-cad is easy to be degraded and has shown Western blotting results similar to the manuscript. In addition, the epitopes of the anti-VE-cad and anti-phosphorylated VE-cad antibodies should be provided in the method.

I’ve added two papers showing the degradation of VE-cadherin using immunoblotting and as references in a newly added sentence in the result. The description on the epitope of VE-cadherin antibody (Santa Cruz) and pY658-VE-cadherin antibody has been added to the method section. Unfortunately, Abcam does not open the information on the epitope of their VE-cadherin antibody (ab33168).

Attachment Submitted filename: responses-final.docx

# Peer review: Decision Letter 2

**DOI:** 10.1371/journal.pone.0302628.r005

**Authors:** You-Yang Zhao

8 Apr 2024

Mexenone protects mice from LPS-induced sepsis by EC barrier stabilization

PONE-D-23-14333R2

Dear Dr. Yun,

We’re pleased to inform you that your manuscript has been judged scientifically suitable for publication and will be formally accepted for publication once it meets all outstanding technical requirements.

Within one week, you’ll receive an e-mail detailing the required amendments. When these have been addressed, you’ll receive a formal acceptance letter and your manuscript will be scheduled for publication.

An invoice will be generated when your article is formally accepted. Please note, if your institution has a publishing partnership with PLOS and your article meets the relevant criteria, all or part of your publication costs will be covered. Please make sure your user information is up-to-date by logging into Editorial Manager at Editorial Manager® and clicking the ‘Update My Information' link at the top of the page. If you have any questions relating to publication charges, please contact our Author Billing department directly at authorbilling@plos.org.

If your institution or institutions have a press office, please notify them about your upcoming paper to help maximize its impact. If they’ll be preparing press materials, please inform our press team as soon as possible -- no later than 48 hours after receiving the formal acceptance. Your manuscript will remain under strict press embargo until 2 pm Eastern Time on the date of publication. For more information, please contact onepress@plos.org.

Kind regards,

You-Yang Zhao

Academic Editor

PLOS ONE

Additional Editor Comments (optional):

Reviewers' comments:

Reviewer's Responses to Questions

**Comments to the Author**

1. If the authors have adequately addressed your comments raised in a previous round of review and you feel that this manuscript is now acceptable for publication, you may indicate that here to bypass the “Comments to the Author” section, enter your conflict of interest statement in the “Confidential to Editor” section, and submit your "Accept" recommendation.

Reviewer #4: All comments have been addressed

**********

2. Is the manuscript technically sound, and do the data support the conclusions?

The manuscript must describe a technically sound piece of scientific research with data that supports the conclusions. Experiments must have been conducted rigorously, with appropriate controls, replication, and sample sizes. The conclusions must be drawn appropriately based on the data presented. 

Reviewer #4: Yes

**********

3. Has the statistical analysis been performed appropriately and rigorously? 

Reviewer #4: Yes

**********

4. Have the authors made all data underlying the findings in their manuscript fully available?

The PLOS Data policy requires authors to make all data underlying the findings described in their manuscript fully available without restriction, with rare exception (please refer to the Data Availability Statement in the manuscript PDF file). The data should be provided as part of the manuscript or its supporting information, or deposited to a public repository. For example, in addition to summary statistics, the data points behind means, medians and variance measures should be available. If there are restrictions on publicly sharing data—e.g. participant privacy or use of data from a third party—those must be specified.

Reviewer #4: Yes

**********

5. Is the manuscript presented in an intelligible fashion and written in standard English?

PLOS ONE does not copyedit accepted manuscripts, so the language in submitted articles must be clear, correct, and unambiguous. Any typographical or grammatical errors should be corrected at revision, so please note any specific errors here.

Reviewer #4: Yes

**********

6. Review Comments to the Author

Please use the space provided to explain your answers to the questions above. You may also include additional comments for the author, including concerns about dual publication, research ethics, or publication ethics. (Please upload your review as an attachment if it exceeds 20,000 characters)

Reviewer #4: All concerns were appropriately answered by the authors and described appropriately in the revised manuscript. The manuscript should now be accepted for publication.

**********

7. PLOS authors have the option to publish the peer review history of their article (what does this mean?). If published, this will include your full peer review and any attached files.

If you choose “no”, your identity will remain anonymous but your review may still be made public.

**Do you want your identity to be public for this peer review?** For information about this choice, including consent withdrawal, please see our Privacy Policy.

Reviewer #4: No

**********

# Editor report: Acceptance letter

**DOI:** 10.1371/journal.pone.0302628.r006

**Authors:** You-Yang Zhao

26 Apr 2024

PONE-D-23-14333R2 

PLOS ONE

Dear Dr. Yun, 

I'm pleased to inform you that your manuscript has been deemed suitable for publication in PLOS ONE. Congratulations! Your manuscript is now being handed over to our production team.

At this stage, our production department will prepare your paper for publication. This includes ensuring the following:

* All references, tables, and figures are properly cited

* All relevant supporting information is included in the manuscript submission,

* There are no issues that prevent the paper from being properly typeset

If revisions are needed, the production department will contact you directly to resolve them. If no revisions are needed, you will receive an email when the publication date has been set. At this time, we do not offer pre-publication proofs to authors during production of the accepted work. Please keep in mind that we are working through a large volume of accepted articles, so please give us a few weeks to review your paper and let you know the next and final steps. 

Lastly, if your institution or institutions have a press office, please let them know about your upcoming paper now to help maximize its impact. If they'll be preparing press materials, please inform our press team within the next 48 hours. Your manuscript will remain under strict press embargo until 2 pm Eastern Time on the date of publication. For more information, please contact onepress@plos.org.

If we can help with anything else, please email us at customercare@plos.org.

Thank you for submitting your work to PLOS ONE and supporting open access. 

Kind regards, 

PLOS ONE Editorial Office Staff

on behalf of

Dr. You-Yang Zhao 

Academic Editor

PLOS ONE
